# Epidemiology of Suicidal Ideation, Planning, and Attempts in School-Going Adolescents in the Wasit Governorate: An Updated Wide-Scale Study

**DOI:** 10.7759/cureus.91456

**Published:** 2025-09-02

**Authors:** Issam S Ismail, Saeed S Hashemi, Yadollah Mehrabi, Hasan A Baiee, Faris H Lami, Salih M Al Hasnawi

**Affiliations:** 1 Department of Epidemiology, School of Public Health and Safety, Shahid Beheshti University of Medical Sciences, Tehran, IRN; 2 Mental Health Department, The Health General Directorate of Wasit Governorate, Ministry of Health, Wasit, IRQ; 3 Department of Public Health and Community Medicine, University of Hilla, Babylon, IRQ; 4 Department of Community Medicine, College of Medicine, Baghdad University, Baghdad, IRQ; 5 Department of Community Medicine, Al-Subtain University for Medical Sciences, Kerbala, IRQ; 6 Department of Psychiatry, Ministry of Health, Baghdad, IRQ

**Keywords:** adolescents, iraq, planning, population based study, secondary schools, suicidal behavior, suicidal ideation and attempt, wasit

## Abstract

Background

Suicide represents a serious global public health challenge and a leading cause of mortality and morbidity in adolescents. This study aimed to investigate the occurrence and some determinants of suicidal ideation, planning, and attempts among school-going adolescents in the Wasit Governorate, Iraq.

Methodology

This was an updated, wide-scale cross-sectional study targeting public governmental middle- and high-school students aged 13 to 18 years. Schools and students were selected using a standardized two-stage cluster sampling design. A pretested Arabic Version of the Iraqi Global School-Based Student Health Survey questionnaire was used in addition to demographic and psychosocial variables, including parental socio-economic status and history of suicidal behaviors among family members and friends. All procedures were performed under ethical standards. The survey data analysis was performed using the primary sampling unit (PSU) and finite population correction (FPC) methods with a baseline univariate analysis and multivariate logistic regression to measure the associated factors.

Results

A total of 1810 students from 42 middle and high schools were included, with a mean age of 15.98 years. Boys formed 50.27% of the study population. The overall 12-month prevalence of suicidal ideation, planning, and attempts was 23.87% (95% CI: 19.65-28.67), 21.90% (95% CI: 18.04-26.32), and 22.37% (95% CI: 18.60-26.65), respectively. In the univariate analysis, suicidal behavior, and thoughts had significant associations with gender, social status, residency, parental conflict, and the presence of suicidal behavior history in family and friends. In the multivariate full-model analysis, the risk factors for suicide attempts were girls (adjusted odds ratio or AOR 1.64; 95% CI; 1.05-2.58), exposure to suicide attempts in family members (AOR 3.36, 95% CI: 1.60-7.06) and friends (AOR 1.99, 95% CI: 1.26-3.15). Suicidal ideation (AOR 11.81, 95% CI: 7.59-18.40) and planning (AOR 8.06, 95% CI: 4.30-15.10) were the most important associated risk factors for suicide attempts.

Conclusion

Suicidal ideation, planning, and attempts are alarmingly prevalent among school-going adolescents in Wasit, particularly among girls and those with a history of suicide attempts in friends and family members. Suicidal ideation and planning strongly relate to suicide attempts. Multi-level and collaborative efforts toward preventing the onset of ideation, planning, and attempts are crucial.

## Introduction

Suicide represents a serious global public health challenge and is a leading cause of mortality and morbidity among adolescents, particularly in low- and middle-income countries. It contributes to more than 700,000 deaths each year globally [1], and the burden is even greater when considering the range of suicidal behaviors, including thoughts, planning, and attempts [2]. Suicide can occur throughout the lifespan and may affect individuals from all social classes [3]. Up to 50% of all mental health conditions begin before the age of 14, and suicide is one of the leading causes of death among adolescents [4].

The occurrence of suicidal ideation, planning, and attempts is far more common than fatalities, and much higher among adolescents than adults [5]. It is estimated that for each suicide, there are between 10 and 30 attempts, and an exponentially greater number of individuals suffer from suicidal ideation [6]. Many researchers have relied on data derived from the Global School-based Student Health Survey (GSHS) to comprehend the suicidal behaviors of in-school adolescents aged 13-17 years [7]. A pooled 12-month prevalence of suicide ideation, plan, and attempt from a recent systematic review and meta-analysis study across 59 low-income and middle-income countries was 16.9%, 17%, and 17%, respectively [8].

There is considerable heterogeneity between countries in the prevalence of suicide ideation, planning, and attempts in adolescents. Most often, the prevalence of suicidal ideation and/or attempts was higher among girls than boys [8-16]. This does not seem to be the case in preadolescent children, where males appear more likely to have suicidal thoughts and behaviors (STBs) than in adolescence [17].

However, the factors that place individuals at risk for suicide are complex and interact with one another [18]. Literature from different settings identified various risk factors that may increase the risk of suicidal behaviors occurring in adolescents. These mainly included the exposure to bullying/cyberbullying and violence [12-14,18-22], family history of suicide [18,23,24], alcohol and substance abuse [6,9,11,12,18,24], tobacco use [12], psychiatric disorders [2,6,9,10,12,14,17,18], weak family and peer relationships [10,12-14,24], and digital technology-related factors, especially problematic use of social media/internet [25], and unprofessional media reporting that sensationalizes suicide incidents, increasing the risk of “copycat” suicides [26].

In addition, the COVID-19 pandemic severely reduced the well-being of young people and put them at increased risk of suicide, substance abuse, and mental health disorders [27]. Furthermore, suicidal ideation is a well‐established risk factor for suicidal attempts [17,28-30].

In Iraq, national and comprehensive studies about suicide and suicidality are nonexistent or scarce [31]. In this context, the first GSHS was carried out in 2012 in middle schools, and the prevalence of suicidal ideation and attempts was 17.37% and 16.67%, respectively [32]. Also, the first national population-based survey on suicide was conducted in 2015/2016, and it indicated that suicide was more common among young people, and the crude rate of suicide per 100,000 population was 1.09 and 1.31 in 2015 and 2016, respectively, with no significant difference between males and females [33].

Over the last five years, the reported suicide incidence rates, derived from annual health statistics reports issued by the Iraqi Ministry of Health, were 1.0, 0.8, 1.7, 1.4, and 1.0 per 100000 population in 2019, 2020, 2021, 2022, and 2023, respectively, with an increasing trend in 2021 and 2022 [34]. The spatial distribution of the reported suicide incidence rate in 2023 showed that the highest incidence rate (1.8 per 100000 population) was in Wasit and Karbala Governorates.

The rising number of suicide cases in Iraq over the last few years is a worrying public health concern that can no longer be ignored. If not addressed, it will take a heavy toll on individuals and communities in the country [35]. Therefore, with the rarity of studies in Wasit Governorate, in this study, we aimed to investigate the prevalence and correlates of suicidal ideation, planning, and suicide attempts in secondary schools in all the six districts of the Governorate.

## Materials and methods

Study design and setting

This was a wide-scale cross-sectional study targeting school students in public governmental intermediate (middle) and preparatory (high) schools in the Wasit Governorate, located about 160 kilometers southeast of Baghdad, the capital of Iraq. The total population was 1,566,789 in 2023 [34]. The Governorate is divided by the Tigris River into the right and left sides.

Study population, sampling method, and sample size

Students aged 13-18 years were selected from the public intermediate and preparatory schools in the governorate by using a standardized two-stage cluster sampling design. In the first stage, the schools were selected based on probability proportional to size sampling. Out of 432 middle and high schools, 42 schools in six districts and 24 sub-districts, comprising both sexes, were selected. These 432 public schools included about 88% of the students in secondary schools in Wasit, as per data derived from the 2023/2024 database. At the second stage, classes were randomly selected from these schools, and all the students in the selected classes were eligible to participate.

The sample size was calculated using the formula, n= (z^2 ^p(1-p))/ d^2^) x deff, [36]. The estimated sample size was 1782 students, considering a 16.6% prevalence (p) of suicide attempts among school students (GSHS data, Iraq 2012) [32], a precision or a margin of error set at 0.02, CI at 95%, a non-response rate of 10.0%, and a 1.2 design effect.

Exclusion criteria

Students in private, vocational, religious, super (super schools for students who achieved high scores >90), and evening schools (evening schools established for students who failed in the public schools and whose ages were older than their peers) were excluded. These were 107 schools with about 32,343 students. Students in these schools formed 12% of the students in the secondary schools in the Governorate.

Measurements and data collection

A standard Arabic version of the Iraqi GSHS questionnaire was used. Questions about dependent variables, suicidal ideation, planning, and attempts were derived from it [37].

- Suicidal ideation: "During the past 12 months, did you ever seriously consider attempting suicide?” The answer to this question was binary (yes, no).

- Suicidal planning: "During the past 12 months, did you make a plan about how you would attempt suicide?" The answer was a binary response (yes, no).

- Suicidal attempt: “During the past 12 months, how many times did you actually attempt suicide?" Response options were zero times, one time, two to three times, four to five times, or greater than or equal to six times. Responses were dichotomized to none (zero times) and greater than or equal to one time (≥one time).

In addition, we asked questions about socio-demographic variables including age, sex, place, marital status, religion (question about religion was derived from the Multiple Indicator Cluster Surveys, United Nations Children's Fund (MICS UNICEF) survey) [38], and the type of school (middle, high). Given that the GSHS survey questionnaire does not include questions on the socio-economic conditions of the parents, we included information on the educational levels and occupations of the parents to capture some socio-economic contributors. Both education and occupation status were categorized using classification guidelines of International Standard Classification of Education (ISCED) 2011 and International Labour Organization (ILO-STAT), respectively [39,40]. Questions about family cohesion, lifetime history of suicide attempts among family and friends, and completed suicide were also included.

Data collection began from 21 April through 19 May 2024. Students were informed about the purpose of the study and its objectives. They gave informed assent (agreement) and reassured that their participation was voluntary. Confidentiality was protected by anonymity, and hey answered privately under the researcher’s supervision without co-supervision from the teachers. Students completed a self-administered questionnaire during one class and recorded their responses on the answer sheet. Pencils were distributed to all students, and after receiving the questionnaire, the pencils were given to students as a simple gift. All data were gathered directly by the chief researcher, except for two schools where another well-trained postgraduate investigator collected them.

Ethical approval

Ethical approval was obtained from the Research Ethics Committees of the School of Public Health and Neuroscience Research Center-Shahid Beheshti University of Medical Sciences (Ethic code: IR.SBMU.PHNS.REC.1402.046). Also, written approval was obtained from the General Directorate of Education in the Wasit Governorate. In addition, formal consent was obtained from all school directors. A written agreement (assent) was obtained from all students (after they read the cover letter that includes the purpose of the study with a confidentiality and privacy process, they could freely decide to tick either agree or disagree to participate). The research involved no more than minimal risk to the subjects with no invasive procedures. Therefore, parental or guardian consent was waived, and all procedures were performed under ethical standards (Council for International Organizations of Medical Sciences (CIOMS) 2016: guidelines 17 and 10) [41].

Statistical analysis

Data was entered, coded, cleaned, and analyzed using IBM SPSS Statistics for Windows, Version 28 (Released 2021; IBM Corp., Armonk, New York, United States) and STATA/MP 18.0 (Stata Corp LLC, College Station, TX, US). Survey data analysis was performed using the primary sampling unit (PSU) and the finite population correction (FPC) methods. Descriptive statistics (frequencies, percentages, and mean ± standard deviation) and inferential statistics (binary logistic regression and multivariate logistic regression analysis with crude and adjusted odds ratio and 95% CI), and the Archer-Lemeshow test for models’ goodness of fit were used [42]. Four statistical models of STBs were developed with all associated independent covariates. Variables with a p-value of ≤0.2 in the univariate analysis were entered into multivariate analysis. A p-value of <0.05 was considered statistically significant. Complete-case analysis (CCA) was used to handle variables with missing data of ≤10% [43].

## Results

Overall prevalence of suicidal ideation, planning, and attempts

A total of 1810 schoolchildren of both sexes from 42 middle and high schools were involved in this large, updated, and wide-scale school-based study to determine the prevalence of STBs in the Wasit Governorate. The response rate was 100% and 98% from schools and students, respectively; 30 students were unwilling to participate. The 12-month prevalence of suicidal ideation, planning, and attempts at least one time was 23.87%, 21.9%, and 22.37%, respectively (Table 1).

**Table 1:** The 12-month prevalence estimates of suicidal behaviors (n=1810) *Percentage of missing values was 3.8%, 2.6%, and 1.5% for suicidal ideation, planning, and attempts, respectively.

Suicide items	Frequency*	Weighted% (95% CI)
Ideation	421	23.87 (19.65-28.67)
Planning	383	21.90 (18.04-26.32)
Attempts	409	22.37 (18.60-26.65)

Univariate analysis

Socio-demographic Details and Type of School

The overall mean age of the children in the sample was 15.98 years (95% CI: 15.36-16.59; range: 12-22 yrs), about half of the students fell within the age group of 15-17 years (n=849). Boys formed 50.27% (n=986) of the study population. Most students were singles (n=1694) and Muslims (n=1773). Students from the Al-Kut district (The Center of the Wasit Governorate) represented 44.74% (n=790) of the total sample, and the majority of students, 81.87% (n=1392), resided in urban areas. Students from high schools formed 59.63% (n=986) of the participants (Table 2). 

**Table 2:** Baseline and univariate analysis of the socio-demographic characteristics of the spectrum of suicide behavior in students (n=1810) The univariate analysis was performed with a binary logistic regression. Categorical variables are reported as frequency (%). The total sum for some variables is different due to missing values. *Age was divided into three levels: early adolescence (12 to 14 years), middle (15-17 years), and late adolescence (18-20 years) [44]. **Categories with a small number of observations were omitted in the univariate analysis.

Person, place, and type of school (frequencies)		Suicidal thoughts and behaviors								
		Ideation		OR (95% CI), p-value	Planning		OR (95% CI), p-value	Attempts		OR (95% CI), p-value
		Yes (n=421)	No (n=1315)		Yes (n=383)	No (n=1374)		Yes (409)	No (1369)	
Age, Mean±SD (years)		16.00±15.91	15.97±11.99	-	16.00±15.71	15.97±12.17	-	16.00±15.74	15.97±12.13	-
Age groups (years)*	12-14 (n=604)	135 (24.5)	439 (75.5)	Ref.	116 (21.4)	470 (78.6)	Ref.	139 (23.6)	456 (76.4)	Ref.
	15-17 (n=849)	182 (20.9)	642 (79.1)	0.82 (0.55-1.21), 0.307	177 (20.8)	655 (79.2)	0.97 (0.65-1.44), 0.860	170 (19.2)	666 (80.8)	0.77 (0.50-1.19), 0.223
	18-20 (n=336)	99 (29.5)	225 (70.5)	1.29 (0.76-2.19), 0.343	86 (25.1)	239 (74.9)	1.23 (0.67-2.26), 0.499	95 (27.4)	238 (72.6)	1.23 (0.71-2.11), 0.452
Sex	Male (n=986)	188 (19.3)	754 (80.7)	Ref.	172 (18.0)	791 (82.0)	Ref.	173 (17.3)	798 (82.7)	Ref.
	Female (n=817)	233 (28.4)	561 (71.6)	1.66 (1.06-2.59), 0.028	211 (25.9)	583 (74.1)	1.59 (1.02-2.48), 0.040	236 (27.5)	571 (72.5)	1.82 (1.19-2.79), 0.007
Marital status**	Single (n=1694)	386 (23.2)	1244 (76.8)	Ref.	348 (21.1)	1300 (78.9)	Ref.	373 (21.6)	1293 (78.4)	Ref.
	Engaged (n=26)	14 (58.4)	11 (41.6)	4.64 (2.04-10.52), 0.001	14 (54.6)	12 (45.4)	4.50 (2.12-9.55), <0.001	14 (55.1)	12 (44.9)	4.45 (1.72-11.6), 0.003
	Married (n=21)	3 (15.1)	15 (84.9)	0.59 (0.18-1.93), 0.373	2 (12.7)	17 (87.3)	0.54 (0.14-2.12), 0.368	4 (24.0)	16 (76.0)	1.15 (0.43-3.04), 0.776
	Divorced/widowed (n=2)	0 (0.0)	2 (100.0)	-	0 (0.0)	2 (100.0)	-	0 (0.0)	2 (100.0)	-
Place/District	Al-Kut (n=790)	206 (26.8)	553 (73.2)	Ref.	188 (25.0)	578 (75.0)	Ref.	192 (23.8)	585 (76.2)	Ref.
	Al-Hay (n=218)	52 (20.6)	159 (79.4)	0.71 (0.34-1.48), 0.349	46 (17.5)	169 (82.5)	0.64 (0.33-1.23), 0.173	46 (16.7)	171 (83.8)	0.64 (0.32-1.27), 0.198
	Al-Numaniyah (n=210)	45 (25.3)	155 (74.7)	0.92 (0.49-1.73), 0.793	37 (20.2)	169 (79.8)	0.76 (0.43-1.34), 0.330	40 (22.1)	168 (77.9)	0.91 (0.46-1.78), 0.778
	Badra (n=152)	32 (21.8)	112 (78.2)	0.76 (0.41-1.41), 0.373	28 (18.8)	117 (81.2)	0.69 (0.29-1.65), 0.397	33 (19.4)	112 (80.6)	0.77 (0.33-1.80), 0.539
	Azizia (n=201)	46 (23.7)	150 (76.3)	0.85 (0.39-1.85), 0.671	48 (24.1)	150 (75.9)	0.95 (0.54-1.70), 0.869	50 (25.0)	150 (75.0)	1.07 (0.54-2.25), 0.839
	Swaira (n=232)	40 (19.5)	186 (80.5)	0.66 (0.35-1.24), 0.190	36 (17.7)	191 (82.3)	0.65 (0.39-1.07), 0.089	48 (21.9)	183 (78.1)	0.90 (0.55-1.47), 0.654
Inhabit	Urban (n=1392)	347 (25.0)	992 (75.0)	Ref.	322 (23.4)	1037 (76.6)	Ref.	335 (23.1)	1037 (76.9)	Ref.
	Rural (n=411)	74 (18.7)	323 (81.3)	0.69 (0.47-1.01), 0.056	61 (15.3)	337 (84.7)	0.59 (0.40-0.88), 0.011	74 (19.1)	332 (80.9)	0.80 (0.51-1.21), 0.269
School type	Middle (n=817)	208 (27.6)	572 (72.4)	Ref.	186 (25.2)	609 (74.8)	Ref.	211 (26.3)	594 (73.7)	Ref.
	High (n=986)	213 (21.4)	743 (78.6)	0.71 (0.45-1.15), 0.157	197 (19.7)	765 (80.3)	0.73 (0.45-1.17), 0.180	198 (19.7)	775 (80.3)	0.69 (0.45-1.06), 0.089
Grade (class)	First (n=314)	67 (20.5)	231 (79.5)	Ref.	55 (16.9)	249 (83.1)	Ref.	65 (18.6)	245 (81.4)	Ref.
	Second (n=503)	141 (31.6)	341 (68.4)	1.79 (0.85-3.78), 0.124	131 (30.0)	360 (70.0)	2.11 (1.21-3.67), 0.010	146 (30.7)	349 (69.3)	1.93 (1.09-3.43), 0.026
	Fourth (n=506)	106 (19.8)	386 (80.2)	0.96 (0.53-1.71), 0.872	99 (19.3)	393 (80.7)	1.18 (0.78-1.78), 0.421	97 (18.9)	401 (81.1)	1.02 (0.60-1.73), 0.944
	Fifth (n=480)	107 (22.9)	357 (77.1)	1.15 (0.63-2.13), 0.644	98 (20.0)	372 (80.0)	1.23 (0.75-2.03), 0.403	101 (20.6)	374 (79.4)	1.13 (0.60-2.14), 0.702

The baseline characteristics in Table 2 also show that the mean age was approximately 16 years for those with and without suicidal ideation, planning, and attempts. Moreover, students aged 18 years or older exhibited a higher prevalence for suicidal ideation, planning, and attempts compared to their younger peers. Girls were more likely to be exposed to suicidal ideation, planning, and attempts than boys, with ORs of 1.66 (95%CI: 1.06-2.59), 1.59 (95%CI: 1.02-2.48), and 1.82 (95%CI: 1.19-2.79), respectively. Students who engaged in relationships reported a higher prevalence of suicidal ideation, planning, and attempts than those who were single or married; they faced approximately four times the risk of suicidal ideation, planning, and attempts compared to the reference category, with odds ratios of 4.64 (95%CI: 2.04-10.52), 4.50 (95%CI: 2.12-9.55), and 4.45 (95%CI: 1.72-11.6), respectively.

The prevalence of suicidal ideation, planning, and attempts varied across the six districts. Schoolchildren residing in urban areas reported a higher occurrence of suicidal ideation, planning, and attempts than those in rural areas, and living in rural areas demonstrated a protective effect against suicidal ideation and planning, with an OR of 0.69 (95%CI: 0.47-1.01) and 0.59 (95%CI: 0.40-0.88), respectively.

Schoolchildren in middle schools reported a higher prevalence of suicidal ideation, planning, and attempts than students in high school, but with no significant association. In addition, students in the second class were at risk of STBs about two times more than students in other classes. Note that a few students in the third and sixth grades were present in schools on the data collection days, and most left schools early to prepare for the final national exams. Hence, they were not included in this research.

Parental Socioeconomic Status

As seen in Table 3, students reported that 56% of their fathers and 74% of their mothers had basic and less than basic educational attainment.

**Table 3:** Parental educational attainment and occupational status (n=1810) The univariate analysis was performed with a binary logistic regression. Categorical variables are reported as frequency (%). The total sum for some variables is different due to missing values. *Categories with a small number of observations were omitted in the univariate analysis.

Parental socio-economic status (frequencies)		Suicide thoughts and behaviors								
		Ideation		OR (95% CI), p-value	Planning		OR (95% CI), p-value	Attempts		OR (95% CI), p-value
		Yes (421)	No (1315)		Yes (383)	No (1374)		Yes (409)	No (1369)	
Fathers' education	Less than basic (n=248)	68 (26.5)	172 (73.5)	Ref.	56 (23.1)	187 (76.9)	Ref.	61 (22.2)	185 (77.8)	Ref.
	Basic (n=727)	157 (22.4)	542 (77.6)	0.80 (0.58-1.10), 0.165	160 (22.6)	555 (77.4)	0.97 (0.74-1.29), 0.842	163 (22.6)	556 (77.4)	1.02 (0.74-1.42), 0.889
	Intermediate (n=241)	60 (25.5)	176 (74.5)	0.95 (0.56-1.62), 0.843	44 (17.2)	187 (82.8)	0.69 (0.45-1.06), 0.086	51 (19.9)	185 (80.1)	0.87 (0.52-1.50), 0.582
	Advanced (n=375)	84 (21.8)	274 (78.2)	0.77 (0.44-1.35), 0.356	81 (22.3)	282 (77.7)	0.96 (0.62-1.48), 0.845	85 (22.8)	284 (77.2)	1.03 (0.68-1.57), 0.880
	Don't know (n=153)	35 (27.4)	113 (72.6)	1.05 (0.61-1.79), 0.870	32 (23.3)	116 (76.7)	1.01 (0.60-1.70), 0.964	32 (23.3)	119 (76.7)	1.06 (0.64-1.77), 0.809
Mothers' education	Less than basic (n=478)	97 (20.5)	364 (79.5)	Ref.	84 (19.5)	378 (80.5)	Ref.	91 (18.0)	382 (82.0)	Ref.
	Basic (n=832)	204 (24.2)	598 (75.8)	1.24 (0.84-1.83), 0.273	189 (21.9)	624 (78.1)	1.16 (0.75-1.79), 0.497	203 (23.4)	618 (76.6)	1.39 (1.00-1.94), 0.049
	Intermediate (n=137)	40 (29.4)	93 (70.6)	1.61 (0.89-2.94), 0.114	35 (24.6)	99 (75.4)	1.35 (0.80-2.27), 0.251	36 (26.0)	97 (74.0)	1.61 (0.93-2.76), 0.086
	Advanced (n=226)	51 (23.5)	164 (76.5)	1.19 (0.73-1.95), 0.477	50 (23.8)	171 (76.2)	1.29 (0.72-2.32), 0.382	52 (26.0)	169 (74.00)	1.44 (0.89-2.35), 0.136
	Don't know (n=104)	22 (23.5)	79 (76.5)	1.34 (0.75-2.37), 0.312	18 (20.0)	84 (80.0)	1.03 (0.60-1.77), 0.912	19 (20.5)	85 (79.5)	1.17 (0.66-2.10), 0.582
Fathers' occupation	Low (n=66)	22 (32.8)	42 (67.2)	Ref.	21 (32.2)	45 (67.8)	Ref.	16 (25.4)	50 (74.6)	Ref.
	Medium (n=417)	88 (24.3)	314 (75.7)	0.66 (0.31-1.39), 0.267	76 (19.1)	332 (80.9)	0.50 (0.26-0.96), 0.038	94 (23.6)	316 (76.4)	0.91 (0.42-1.95), 0.795
	High (n=256)	64 (26.0)	178 (74.0)	0.72 (0.34-1.51), 0.373	66 (28.5)	179 (71.5)	0.84 (0.43-1.65), 0.601	65 (23.8)	185 (76.2)	0.92 (0.43-1.98), 0.821
	Armed forces (n=193)	52 (22.8)	135 (77.2)	0.61 (0.31-1.18), 0.135	44 (20.4)	141 (79.6)	0.54 (0.31-0.93), 0.028	47 (23.1)	145 (76.9)	0.88 (0.42-1.85), 0.732
	Unspecified jobs (n=220)	47 (22.5)	166 (77.5)	0.59 (0.25-1.41), 0.231	42 (19.4)	174 (80.6)	0.51 (0.27-0.96), 0.038	48 (20.8)	168 (79.2)	0.79 (0.35-1.71), 0.511
	Retired (n=41)	11 (25.8)	29 (74.2)	0.71 (0.23-2.22), 0.550	11 (27.0)	30 (73.0)	0.78 (0.27-2.28), 0.644	10 (26.1)	31 (73.9)	1.04 (0.34-3.15), 0.948
	Unspecified free works (n=375)	84 (22.6)	278 (77.4)	0.560 (0.28-1.26), 0.168	75 (21.5)	293 (78.5)	0.58 (0.26-1.28), 0.172	79 (20.9)	292 (79.1)	0.78 (0.34-1.78), 0.539
	Dead & martyrs (n=68)	16 (28.5)	50 (71.5)	0.82 (0.37-1.78), 0.603	13 (19.3)	54 (80.7)	0.51 (0.25-1.04), 0.064	15 (25.4)	53 (74.6)	0.10 (0.41-2.42), 0.933
	Don't work (n=46)	12 (24.4)	32 (75.6)	0.66 (0.30-1.45), 0.292	10 (21.4)	34 (78.6)	0.57 (0.25-1.30), 0.176	9 (13.2)	36 (86.8)	0.45 (0.13-1.53), 0.192
	Students (n=3)*	0 (0.0)	3 (100.0)	-	0 (0.0)	3 (100.0)	-	1 (30.1)	2 (69.9)	-
	Don't know (n=3)*	2 (66.7)	1 (33.3)	-	1 (34.2)	2 (65.8)	-	1 (34.2)	2 (65.8)	-
Mothers' occupation	Low (n=3)*	0 (0.0)	3 (100.0)	-	0 (0.0)	3 (100.0)	-	1 (39.0)	2 (61.0)	-
	Medium (n=25)	7 (28.0)	18 (77.9)	0.94 (0.37-2.38), 0.884	6 (24.9)	19 (75.1)	1.24 (0.43-3.52), 0.685	3 (7.6)	22 (92.4)	0.30 (0.06-1.44), 0.127
	High (n=171)	43 (29.3)	120 (70.7)	1.36 (0.80-2.34), 0.252	39 (26.6)	127 (73.4)	1.44 (0.77-2.35), 0.283	45 (30.2)	122 (69.8)	1.55 (0.89-2.70), 0.115
	Housewife (n=1355)	312 (23.3)	999 (76.7)	Ref.	289 (21.2)	1032 (78.8)	Ref.	307 (21.8)	1032 (78.2)	Ref.
	Don't work (n=71)	14 (22.4)	52 (77.6)	0.95 (0.45-1.00), 0.882	13 (22.8)	58 (77.2)	1.10 (0.56-2.18), 0.777	15 (23.4)	56 (76.6)	1.10 (0.59-2.05), 0.753
	Unspecified jobs (n=46)	10 (23.1)	35 (76.9)	0.99 (0.42-2.30), 0.973	7 (16.5)	39 (83.5)	0.73 (0.34-1.56), 0.407	8 (17.9)	38 (82.1)	0.78 (0.39-1.59), 0.491
	Unspecified free works (n=17)	5 (21.6)	12 (78.4)	0.90 (0.30-2.76), 0.856	3 (14.9)	14 (85.1)	0.65 (0.17-2.55), 0.527	5 (25.8)	12 (74.2)	1.25 (0.36-4.37), 0.718
	Dead (n=6) *	3 (50.5)	3 (49.5)	-	3 (49.5)	3 (50.5)	-	3 (49.5)	3 (50.5)	-
	Don't know (n=3)*	2 (66.7)	1 (33.3)	-	1 (33.3)	2 (66.7)	-	1 (33.3)	2 (66.7)	-
	Retired (n=2)*	-	-	-	1 (100.0)	0 (0.0)	-	1 (100.0)	0 (0.0)	-
	Postgraduate student (n=1)*	0 (0.0)	1 (100.0)	-	0 (0.0)	1 (100.0)	-	0 (0.0)	1 (100.0)	-

About 9% and 6% of participants didn’t know the academic level of their fathers and mothers, respectively. The data summary of about 250 job titles and occupations showed that 60% of fathers had a medium occupation level, unspecified jobs, and unspecified free work, and 80% of mothers were housewives. Data analysis of the parental socioeconomic status revealed that prevalence of suicidal ideation, planning, and attempts were slightly differently across various backgrounds.

Parental Conflict and a Lifetime History of Suicide in Family and Friends

As shown in Table 4, students reported that in 90.3% (n=1575) cases, their parents had a good relationship within the family, 7.3% (n=128) had parental relationship discord, and in 2.4% (n=42) cases, parents were either divorced or separated.

**Table 4:** Family cohesion and lifetime history of suicide among family and friends (n=1810) The univariate analysis was performed with a binary logistic regression. Categorical variables are reported as frequency (%). The total sum for some variables is different due to missing values.

Family and friends' history of suicide (frequencies)		Suicide thoughts and behaviors								
		Ideation		OR (95% CI), p-value	Planning		OR (95% CI), p-value	Attempts		OR (95% CI), p-value
		Yes (n=421)	No (n=1315)		Yes (n=383)	No (n=1374)		Yes (n=409)	No (n=1369)	
Parental relationship	Good (n=1575)	330 (21.6)	1186 (78.4)	Ref.	294 (19.4)	1239 (80.6)	Ref.	323 (20.2)	1231 (79.8)	Ref.
	Conflict (n=128)	62 (44.9)	64 (55.1)	2.96 (1.97-4.42), <0.001	60 (44.7)	65 (55.3)	3.36 (2.13-5.31), <0.001	55 (42.5)	69 (57.5)	2.92 (1.95-4.37), <0.001
	Separated/divorced (n=42)	15 (36.2)	25 (63.8)	2.06 (1.14-3.72), 0.018	15 (38.9)	27 (61.1)	2.64 (1.56-4.48), 0.001	16 (35.1)	26 (64.9)	2.14 (1.14-4.00), 0.019
Lifetime history of suicide in family	No (n=1605)	346 (21.7)	1210 (78.3)	Ref.	316 (20.1)	1262 (79.9)	Ref.	326 (19.5)	1268 (80.5)	Ref.
	Unsuccessful attempt (n=143)	66 (47.3)	74 (52.7)	3.24 (1.95-5.38), <0.001	57 (43.1)	80 (56.9)	3.01 (1.99-4.55), <0.001	71 (51.7)	71 (48.3)	4.41 (2.98-6.54), <0.001
	Completed suicide (n=17)	5 (42.0)	11 (58.0)	2.61 (0.82-8.37), 0.103	7 (38.2)	10 (61.8)	2.46 (0.78-7.73), 0.121	6 (37.3)	11 (62.7)	2.447 (0.73-8.16), 0.140
Lifetime history of suicide among friends	No (n=1430)	270 (19.5)	1117 (80.5)	Ref.	242 (18.0)	1164 (82.0)	Ref.	253 (17.4)	1169 (82.6)	Ref.
	Unsuccessful attempt (n=215)	104 (48.0)	105 (52.0)	3.81 (2.89-5.02), <0.001	96 (43.7)	113 (56.3)	3.53 (2.64-4.72), <0.001	108 (48.4)	105 (51.6)	4.44 (3.35-5.89), <0.001
	Completed suicide (n=95)	33 (34.2)	60 (65.8)	2.15 (1.26-3.67), 0.006	30 (30.6)	63 (69.4)	2.00 (1.04-3.84), 0.037	33 (36.0)	60 (64.0)	2.67 (1.47-4.85), 0.002
Ideation	Yes (n=421)	-	-	-	-	-	-	315 (74.8)	102 (25.2)	49.37 (33.40-72.97), <0.001
	No (n=1315)	-	-	-	-	-	-	78 (5.7)	1235 (94.3)	Ref.
Planning	Yes (n=383)	-	-	-	-	-	-	299 (76.0)	82 (24.0)	39.94 (26.63-59.92), <0.001
	No (n=1374)	-	-	-	-	-	-	104 (7.4)	1267 (92.6)	Ref.

Students who lived in a family with a conflict atmosphere were at about three times greater risk of suicidal ideation, planning, and attempts compared to students who lived in an intact atmosphere, with an OR (95% CI) of 2.96 (1.97-4.42), 3.36 (2.13-5.31), and 2.92 (1.95-4.37), respectively. Also, students with parents who were separated or divorced were more likely to be exposed to suicidal ideation, planning, and attempts than students who lived in an intact family.

The lifetime prevalence of attempted or completed suicide among family members and friends was 9.1% (n=160) and 17.8% (n=310), respectively. Students who had a history of suicide attempts or completed suicide among family and friends were highly vulnerable to suicidality compared to those who have no history of suicidality. Finally, about 75% of students with suicidal ideation and planning reported suicide attempts.

Multivariate analysis

Table 5 shows the multivariate analysis of four statistical models.

**Table 5:** Risk factors associated with suicidal ideation, planning, and attempts (n=1810) The data analysis was performed with a multivariable logistic regression.

Factors	Categories	Statistical models/Adjusted Odds ratio (95% CI), p-value			
		Suicidal ideation (SI)	Suicidal planning (SP)	Suicidal attempt (SA)	Full model (Attempt)
Sex	Boys	Ref.	Ref.	Ref.	Ref.
	Girls	1.52 (1.03-2.24), 0.038	1.47 (1.02-2.13), 0.042	1.75 (1.23-2.48), 0.003	1.64 (1.05-2.58), 0.032
Marital status	Single	Ref.	Ref.	Ref.	Ref.
	Engaged	4.88 (2.11-11.32), <0.001	4.57 (2.21-9.44), <0.001	4.82 (1.83-12.71), 0.002	1.75 (0.48-6.33), 0.385
	Married	1.01 (0.24-4.26), 0.989	0.96 (0.320-4.63), 0.954	2.15 (0.67-6.97), 0.193	5.37 (1.14-25.33), 0.035
Inhabit	Urban, Ref.	0.75 (0.46-1.21), 0.226	0.61 (0.39-0.94), 0.025	Not in the equation	Not in the equation
	Rural				
School type	Middle, Ref.	0.63 (0.43-0.92), 0.017	0.65 (0.46-0.92), 0.017	0.65 (0.48-0.88), 0.006	Not in the equation
	High				
Family cohesion	Good relationship	Ref.	Ref.	Ref.	Ref.
	Conflict	2.44 (1.65-3.59), <0.001	2.84 (1.78-4.53), <0.001	2.42 (1.62-3.61), <0.001	1.26 (0.57-2.77), 0.563
	Separated/ divorced	1.57 (0.77-3.21), 0.212	2.13 (1.12-4.04), 0.022	1.63 (0.73-3.63), 0.227	0.78(0.16-3.79), 0.752
Lifetime history of suicide among family	No	Ref.	Ref.	Ref.	Ref.
	Unsuccessful attempt	2.04 (1.23-3.69), 0.020	1.76 (1.060-2.91), 0.031	2.71 (1.69-4.40), <0.001	3.36 (1.60-7.06), 0.002
	Committed suicide	2.60 (0.88-7.64), 0.082	2.47 (0.770-7.90), 0.125	2.16 (0.68-6.84), 0.186	1.05 (0.08-13.84), 0.970
Lifetime history of suicide among friends	No	Ref.	Ref.	Ref.	Ref.
	Unsuccessful attempt	2.61 (1.74-3.91), <0.001	2.79 (1.98-3.95), <0.001	3.08 (2.06-4.62), <0.001	1.99 (1.26-3.15), 0.004
	Committed suicide	2.61 (1.52-4.46), 0.001	2.32 (1.15-4.70), 0.020	2.98 (1.60-5.55), 0.001	1.98 (1.03-3.83), 0.042
Suicidal ideation	No	-	-	-	Ref.
	Yes	-	-	-	11.81 (7.59-18.40), <0.001
Suicidal planning	No	-	-	-	Ref.
	Yes	-	-	-	8.06 (4.30-15.10), <0.001
Archer-Lemeshow test (Sig.)		0.152	0.898	0.888	0.703

The details of the models are given below:

1. In the suicidal ideation model, the dependent variable was suicidal ideation, and the associated risk factors included: girls (adjusted odds ratios (AOR) 1.52), students who were in the engagement period (AOR 4.88), students living in an environment with parental conflict (AOR 2.44), the presence of a family history of suicide attempts (AOR 2.04), history of suicide attempts among friends (AOR 2.61), and completed suicide (AOR 2.61). High school students showed a protective effect compared to middle school students (AOR 0.63).

2. In the suicidal planning model, the dependent variable was suicidal planning, and the associated covariates included: girls (AOR 1.47), students in an engagement period (AOR 4.57), parental conflict (AOR 2.84), parents who are separated/ divorced (AOR 2.13), exposure to suicide attempts within the family (AOR 1.76), and exposure to suicide attempts and completed suicides among peers (AOR 2.79) and (AOR 2.32), respectively. Rural inhabitants and high school students exhibit protective effects, with AORs of 0.61 and 0.65, respectively.

3. In the suicide attempts model, the dependent variable were suicide attempts. Students at risk for suicide attempts included girls (AOR 1.75), those in an engagement period (AOR 4.82), those living in non-intact families (AOR 2.42), those with a family history of suicide attempts (AOR 2.71), and those whose peers had a history of suicide attempts and suicide (AOR 3.08), and (AOR 2.98). High school students exhibited protective effects (AOR 65).

4. The last model was a full model that incorporated suicidal ideation and suicidal planning into the suicidal attempts model. The identified risk factors for suicidal attempts included girls (AOR 1.64), individuals with a family history of suicide attempts (AOR 3.36), and peers with a history of suicide attempts (AOR 1.99) and completed suicide (AOR 1.98), alongside suicidal ideation (AOR 11.81) and planning (AOR 8.06). The Archer-Lemeshow test indicated that the goodness of fit for all models was acceptable.

## Discussion

Mental health problems, including suicide, are an emerging public health priority that takes a heavy toll in the second decade of life [45]. Adolescents comprise 22.9% and 23.1% of the Iraqi and Wasit populations, respectively [46]. It is vital to address the main threats to their health to achieve the Sustainable Development Goals (SDG) targets [47].

This is the first large, wide-scale, and updated study about suicidality among schoolchildren in the Wasit Governorate and at the country level. The key findings of this population-based epidemiological study demonstrate a high prevalence of suicidal ideation, planning, and attempts in middle and high schools. It indicated a growing concern compared to the estimates over the past two decades in the Wasit Governorate and at the national level.

In a large representative sample of secondary school students (n=1067) in Al-Kut city in 2007, researchers found that 8.9% of students reported suicidal thoughts and behavior [48]. Al-Nasrawi MS. et al. revealed a high prevalence of suicidal ideation (32.1%) in middle schools (n=900) in the Kerbala Governorate in 2011 (MSc thesis in Community Health: Al-Nasrawi MS, Al-Janabi NM, Abed BK. School-Based Students' Health Behaviors Survey in Intermediate Schools in Karbala City, 2011). Our estimation is higher than the reported prevalence in the GSHS 2012 [32]. In a study conducted in 2017, Baiee et al. found that 49.5% of high school students in the fourth and fifth classes in Hilla City/Babylon had suicidal ideation in the last 24 months [49]. This was much higher than our findings. In Al-Shirqat City, Salah Eldin Governorate, Salem et al. (2019) reported that the one-year prevalence of suicidal ideation and attempts in a sample of adolescents (n=840) attending primary health care centers was 16.8% and 14%, respectively [50].

Nevertheless, the current prevalence of suicidal ideation, planning, and attempt is higher than its pooled prevalence among schoolchildren from 59 different countries, but it falls within the range of (0.2% to 61% for suicidal ideation), (0.9% to 41% for suicidal planning), and (3% to 33% for suicide attempt) [8]. Our estimates were higher by about 3% in comparison to the pooled prevalence from Iranian school-going adolescents for the period between 1988 and 2023, where the prevalence of suicide ideation and attempt was 21% and 18%, respectively [51]. Also, the current prevalence was higher than the pooled prevalence of the overall suicidal ideation, suicide plans, and suicide attempts in Chinese children and adolescents below 18 years old, reported at 15.4% (95% CI (14.3,16.6)), 6.4% (95% CI (5.5, 7.4)) and 3.5% (95% CI (3.1, 4.1)), respectively [52].

In addition, the current estimates were higher than the recently reported prevalence of suicide ideation, planning, and attempt among school-aged youth in the USA, which was at 20%, 16%, and 9%, respectively. The same increasing trend was observed, consistent with a 10-year data summary of YRBS that showed significant rises in suicidal ideation, planning, and attempts among high school students in the USA [53], considering racial, ethnic, and demographic differences between the two societies. It is worth noting that these estimations reflect reporting of in-school adolescents. Studies are needed to investigate the occurrence of STBs in adolescents who have no education and who drop out of school in the Wasit Governorate.

Accordingly, for both suicides and suicide attempts, improved availability and quality of data from vital registration, hospital-based systems, and periodic surveys are required for effective suicide prevention [26].

Risk factors for suicidal ideation, planning, and attempt

In the baseline univariate analysis, the contributing demographic factors for STBs included girls, engagement stage, and living in an urban setting. Suicidal ideation, planning, and attempts were prevalent across all age groups, but students in late adolescence were more likely to engage in suicidal thoughts and attempts than students in early and middle adolescence. The same pattern was reported from global evidence in a recent meta-analysis [8]. The most consistently reported pattern was that the risk of the first onset of suicidal behavior increases significantly at the start of adolescence (12 years), peaks at the age of 16 years, and remains elevated till the early 20s [2].

The STBs exhibited significant geographic disparities, with higher prevalence in urban areas. Globally, there are frequently large disparities in suicide occurrence between urban and rural areas, and the reasons may include social isolation, greater difficulty in detecting warning signs, limited access to health facilities and clinics, and lower levels of education [18,54].

In the Middle East Region and Turkey, urban residents generally have higher suicide rates than rural residents, according to studies from Iran, Turkey, and Egypt [55-57]. In Mongolia, the GSHS reported that the higher prevalence of suicidal ideation was seen in urban schoolchildren [58]. The case in Iraq seems similar to situations in Eastern Mediterranean countries. Results from a recent systematic review study indicate that the suicide rates are higher in urban than in rural communities [59]. However, one study from the Al-Diwaniyah Governorate in Iraq reported contradictory results, where the suicide attempts and complete suicide were similarly distributed to both urban and rural settings, except for the remote rural areas, which still retained a degree of cohesion in social relations [60]. This shows that Iraq still has a significant data gap due to an underdeveloped surveillance system and a shortage of related research [61].

Although there was variation in the prevalence of suicidal ideation, planning, attempts across different socioeconomic backgrounds, the significance of educational attainment and occupation in reporting them could not be confirmed. This may reveal that suicidal ideation, planning, attempts can occur in young people regardless of socioeconomic situation, or there is an indirect effect of the socioeconomic situation on the suicidal ideation, planning, attempts among adolescents. In this regard, we don’t have the socioeconomic situation of the parents in the whole sample; about one-third of students reported unspecified jobs and free work in the case of their fathers, and 15% of participants didn’t know the educational level of their parents. The relevance of socioeconomic factors in suicide attempts and complete suicide was reported in the local studies of human sciences alongside sociocultural changes, life stresses, globalization, weak religious restraint, and psychiatric disorders [62-65]. For example, a study in the Al-Diwaniyah Governorate investigated 41 cases of suicide in 2017 and found that the common causes of suicide were mental disorders and family problems that arise from economic difficulties and being preoccupied with and influenced by everything published on social media [64]. Indeed, behavioral and socioeconomic problems may induce psychological stress that predisposes individuals to suicidal behavior. 

Other risk factors for suicidal ideation, planning, attempts included stressful events that involved social relationships, especially a family history of suicidal acts, parental conflict, and history of suicide attempts and death among friends. Furthermore, suicidal ideation and planning were the main risk factors for suicide attempts.

In the multivariate analysis, girls were 1.5 times more at risk for suicidal ideation, planning, and attempts than boys, and this was important even after adjusting for other risk factors. This is consistent with studies conducted in different countries [8-12, 14-16, 66]. However, results from a meta-analysis, involving 397,299 adolescents, indicated that the prevalence of suicidal ideation was significantly higher among girls than boys. In contrast, attempts did not differ by age or sex [13]. The “gender paradox” in youth, where suicide prevalence is higher in males but suicide ideation and attempts are more common in females, underscores the need for inclusive interventions that consider both genetic vulnerabilities and sex/gender differences [13,67].

The courtship period was the main social determinant in the suicidal ideation, suicide planning, suicide attempts models. Nevertheless, this factor was removed in the full model. Soller B [68] found that inauthenticity in a romantic relationship was positively associated with severe depression, suicide ideation, and suicide attempts, but only for girls. Studies are needed to determine the impact of the pre-marriage period on STBs in our society.

Although the odds of suicidal ideation and attempts were higher in the middle school than in high school, this was not significant after adjusting for other risk factors in the full model. This issue has been reported in different studies [7,69]. Accordingly, there is still a need to investigate this high prevalence of suicidality among middle-school students, including older students with grade repetition.

Evidence from different settings proves the association between family conflict and the poor mental health and STBs in students [10,12-14,24,70]. In this study, students who lived in homes with continuous parental problems were two times more likely to report suicidal ideation, planning, and attempts than those students who lived in intact families.

Exposure to self-harm and suicide of others (family and friends) is associated with adolescent suicidality [2,71,72]. In this study, students who reported a history of family and peer suicide were more at risk for suicidal ideation, planning, and attempts. This may be interpreted by the mechanisms underlying the social contagion of behaviors, including the self-harm seen in others, which may provide a behavioral model for at-risk individuals, thereby increasing the probability that thoughts are acted upon [73]. Also, vulnerable people might cluster together, leading to shared stressors, and self-harm, then induced by a shared stressful event rather than a modeling-type response [74]. Suicidal ideation and planning were the prominent contributing factors to suicide attempts, and this finding is consistent with a lot of evidence from meta-analyses and primary studies [12,19,28-30,67]. Nevertheless, suicide is often thought to occur along a continuous sequence, i.e., ideation precedes plans, which precede attempts [8]. However, evidence suggests that this is not always the case, with some individuals directly engaging in suicidal behaviors without going through other phases such as ideation [75].

Additionally, suicide attempts in adolescents carry a long-term risk. In a large longitudinal birth cohort study, those who made a suicide attempt before the age of 18 years were 18 times more likely to make a suicide attempt between the ages of 18 and 25 [76].

Finally, in the literature, there are tens of bio-psychosocial, behavioral, and economic attributes related to suicide ideation and attempts in adolescents. More comprehensive studies are needed to explain this phenomenon. Accordingly, nowadays, the Qurban phenomenon, a deviant religious ideology that adopts the sacrifice of a person or a group of people (by utilizing suicide) from time to time as part of belonging and love for a holy person, has spread among young people in limited areas in Iraq, including the Wasit Governorate. National Security has arrested a group of these deviants [77]. Qualitative studies are needed to clarify the contributing factors to this serious phenomenon and its effect on the health of adolescents in Wasit.

Strengths and limitations

This was a representative, wide-scale study conducted in a short period and covered all the Wasit Governorate districts. It was an updated large-scale study that offered an important extension of prior literature. Three main limitations to the conclusion drawn are noted. First, the data was cross-sectional, making it impossible to determine the direction of association between variables or to determine causal relationships. Second, self-reporting of behavior makes the data inherently subject to recall bias, and due to the sensitive nature of questions, self-reporting may introduce under- or over-reporting bias. Third, data collection was conducted at the end of the academic year, so the occurrence of STB may have been influenced and induced by the stress of preparing for the final exam and failure to pass the exam at the end of the year. Continuous monitoring and surveillance is needed to observe the trend of STBs over time, and future research, preferably with longitudinal studies and case-control designs, to examine various covariates, is crucial.

## Conclusions

The prevalence of suicidal ideation, planning, and attempts among schoolchildren in Wasit is alarming. Fixed risk factors include female students and having a history of suicidal attempts among peers and family members. Suicidal ideation and planning strongly relate to suicide attempts. Multi-level and collaborative efforts toward preventing the onset of suicide ideation, planning, and attempts are crucial. More importantly, it is necessary to promote socio-emotional life skills in adolescents and to establish specific support for at-risk students.
